# Phylo-Movies: animating phylogenetic trees from sliding-window analyses

**DOI:** 10.1093/molbev/msag194

**Published:** 2026-08-12

**Authors:** Enes Sakalli, Simon Haendeler, Arndt von Haeseler, Heiko A Schmidt

**Affiliations:** Max Perutz Labs, Vienna BioCenter, 1030 Vienna, Austria; University of Vienna, Vienna, Austria; Vienna BioCenter PhD Program, A Doctoral School of the University of Vienna and Medical University of Vienna, Vienna 1030, Austria; Ludwig Boltzmann Institute for Network Medicine, University of Vienna, Vienna, Austria; Digital R&D, Sanofi, 240 Richmond St W, Toronto, ON M5V 2C5, Canada; Ludwig Boltzmann Institute for Network Medicine, University of Vienna, Vienna, Austria; Max Perutz Labs, Vienna BioCenter, 1030 Vienna, Austria; University of Vienna, Vienna, Austria

**Keywords:** animation, recombination, rogue taxa, tree visualization, phylogenetic tree sequence, topological change, SPR move

## Abstract

Sliding-window phylogenetic analyses of multiple sequence alignments generate sequences of phylogenetic trees that can reveal phylogenetic conflict along genomes, as can be caused by recombination and other sources; yet, comparing trees across genomic windows remains challenging. Phylo-Movies is a browser-based tool—also available as a standalone desktop application—that decomposes topological differences between consecutive phylogenetic trees into interpretable rooted subtree prune-and-regraft moves and animates these transformations. We illustrate its use in two contexts: localizing candidate recombination breakpoints in norovirus genomes, where taxa change from polymerase-genotype-associated to capsid-genotype-associated placements at the ORF1/ORF2 junction, and exploring candidate rogue taxa that change position across bootstrap replicates. Phylo-Movies complements quantitative measures such as Robinson–Foulds distances, split-frequency support values, and rogue-taxon scores by showing which subtrees move and how their source and target placements differ. The animations also provide an intuitive educational tool for teaching and workshops. Phylo-Movies is freely available at https://enesberksakalli.github.io/phylo-movies/, with source code at https://github.com/enesBerkSakalli/phylo-movies, and demonstration videos at https://vimeo.com/1199476378, https://vimeo.com/1199476382, https://vimeo.com/1199476534, https://vimeo.com/1199487394, and https://vimeo.com/1199495473.

## Introduction

Ideally, whole-genome phylogenetic inference would recover a single evolutionary history for an entire genome. In practice, genomes may contain regions with different histories. For example, if two related viruses infect a single cell, during genome amplification RNA-dependent RNA polymerase can change the template from one viral genome to the other, commonly referred to as template switching (Simon-Lorière and Holmes 2011). This process links parts of the coinfecting genomes, generating a hybrid virus variant. The different regions of this recombinant genome now reflect the different evolutionary histories of their origin. Similar effects occur via lateral gene transfer between bacteria (Ochman et al. 2000) or crossover during meiosis in eukaryotes (Hunter 2015).

**Figure 1:**
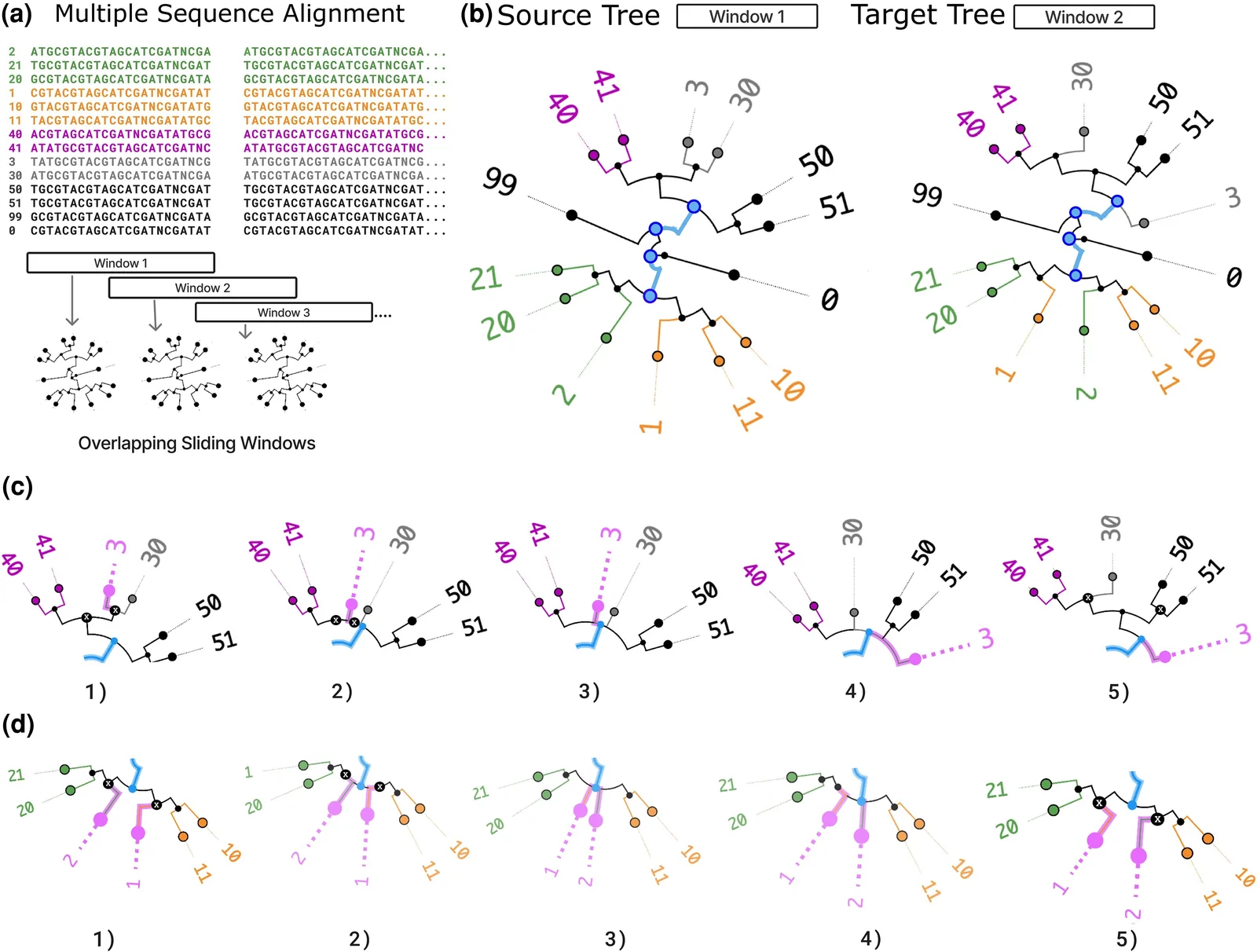
a) A genomic alignment is divided into overlapping sliding windows of defined length and step size. For each window, a phylogenetic tree is reconstructed, generating a sequence of trees for Phylo-Movies. b) Two consecutive trees (windows 1 and 2) are shown as source and target trees. Sequences and corresponding tree nodes are colored according to subtrees defined in the source tree. Two sequential topological changes (upper and lower parts) are illustrated in (c) and (d). Image (c) illustrates the SPR decomposition for one moving leaf, while image (d) shows a more complex case with two independent rearrangements; both examples are described in the “How to animate topological changes between trees” section.

Recombination between highly divergent sequences may disrupt essential gene functions, so successful recombinants typically arise from homologous regions where sequence and gene order are conserved. Consequently, recombinant genomes usually retain enough similarity to be captured in a single multiple sequence alignment (MSA). However, treating such an alignment as a single evolutionary unit can obscure conflicting phylogenetic signals (Posada and Crandall 2002). Sliding-window approaches address this problem by reconstructing a phylogenetic tree for each successive, overlapping window along the alignment (Fig. 1a and b; cf. also Salminen et al. 1995; Lole et al. 1999). Because each window samples only a short genomic segment of the alignment, its tree reflects the local evolutionary history of that region alone. Where no recombination has occurred, consecutive windows yield similar or identical tree topologies (i.e. branching structures independent of branch lengths); where recombination has occurred, the topology changes reflecting ancestry of the recombinant segment, and the genomic position at which this shift occurs approximates the location of a recombination breakpoint.

This approach requires sufficient sequence divergence: recombination between nearly identical parents may leave the tree structure unchanged and thus escape detection.

Several tools exist to analyze recombinant alignments. RDP5 (Martin et al. 2021), SimPlot (Lole et al. 1999), SimPlot++ (Samson et al. 2022), and TOPALi (Milne et al. 2004) focus primarily on detecting recombinants and locating breakpoints. Tree House Explorer takes a complementary approach, showing the distribution of tree topologies across genomic windows in a genome browser (Harris et al. 2022). However, these tools do not visualize how trees from consecutive windows relate to each other, leaving users unable to identify which subtrees move, where they move, or how their source and target placements differ.

The scale of modern datasets compounds this challenge. Small sliding-window step sizes generate hundreds or thousands of largely redundant trees that must be inspected for subtle rearrangements, whereas larger step sizes can produce abrupt topological changes between consecutive windows, making it difficult to track which subtrees change position. For example, sliding-window analysis of a 10-kb HIV genome with a 10-nt step produces approximately 1,000 trees (Abecasis et al. 2007). Identifying the windows where tree rearrangements occur requires tracing branching structures across hundreds of trees, a task poorly suited to static visualization (Graham and Kennedy 2009).

To address these challenges, we developed Phylo-Movies, which animates topological rearrangements between consecutive phylogenetic trees. Users may supply a precomputed tree sequence inferred with their preferred software, or upload an MSA for rapid exploratory tree generation using the IQ-TREE fast-search option (Minh et al. 2020) by default, with FastTree (Price et al. 2010) available as an alternative, and user-defined window and step sizes. For final analyses, users can import ordered tree files inferred externally with IQ-TREE, RAxML, or another preferred phylogenetic program. Trees can be rooted using either an outgroup or midpoint rooting. Phylo-Movies visualizes topological changes by representing the transition between two consecutive trees as a series of rooted subtree prune-and-regraft (SPR) moves. An SPR move prunes a subtree from one location in a rooted tree and regrafts it onto another branch, yielding a new topology after a single rearrangement (Bordewich and Semple 2005). Each SPR move is animated with the affected subtree highlighted, allowing regions of topological change within the tree sequence to be identified. We next describe the algorithmic framework underlying the animation and present two use cases.

## The Phylo-Movies approach

### How to animate topological changes between trees

Phylo-Movies highlights and animates topological changes within a sequence of phylogenetic trees. Visualization requires rooted trees, which can be obtained using either an outgroup or midpoint rooting (Edwards 2019). Trees are displayed using a circular layout (cf. Felsenstein 2004, Chapter 36). This layout was chosen because it keeps moving subtrees visually traceable during animation.

To visualize the whole sequence of trees, topological changes are animated between each pair of consecutive trees, treating the first tree as the source and the second tree as the target (cf. Fig. 1b). To define this transformation, we use the concepts of splits and consensus trees (cf. Bryant 1997).

Every internal branch defines a (nontrivial) split that partitions the leaves into two subsets. Comparing the source and target trees identifies three categories of splits: splits shared by both trees, splits unique to the source tree, and splits unique to the target tree. Please note, the Robinson–Foulds (RF) distance between the two trees is defined as the number of splits unique to the source plus the number unique to the target tree (Robinson and Foulds 1979, 1981).

To visualize the transition from the source tree to the target tree, Phylo-Movies uses the strict consensus tree as intermediate, which contains only the splits present in both trees, with branch lengths set to the mean of the corresponding values in the source and target trees. The transformation is organized around a *pivot edge*, defined as a branch shared by both trees whose immediate descendant branches differ between the source tree and the target tree (rf. the blue edges in Fig. 1b–d). If multiple pivot edges are identified, Phylo-Movies processes the corresponding local conflicts sequentially.

This process is shown in the workflow in Fig. 1. From the alignment in Fig. 1a, window subalignments are extracted to reconstruct the trees in Fig. 1b. Figure 1c shows a topological rearrangement in the upper part of the trees, namely the SPR move of *leaf 3* (marked magenta). In the source tree (Fig. 1c, tree 1), leaf 3 is sister to leaf 30. To reach the target tree topology (Fig. 1c, tree 5), where leaf 3 is placed with the subtree containing leaves 30, 40, 41, 50, and 51, the algorithm identifies the *pivot edge* (thick blue edges in Fig. 1) and performs the rearrangement relative to it.

The transformation begins by shrinking to zero the branch leading to the subtree of leaves 3 and 30, as well as the branch defining the subtree containing 3, 30, 40, and 41 (marked by *x* in trees 1+2 in Fig. 1c). Both branches correspond to splits unique to the source tree and remain visible in tree 2 as black *x* markers. These now zero-length branches are then removed, yielding the intermediate strict consensus tree (tree 3 in Fig. 1c). Subsequently, leaf 3 moves to its correct position within the multifurcation (tree 4 in Fig. 1c), and finally, the two new branches unique to the target tree are inserted and expanded in tree 5 in Fig. 1c: the branch defining subtree (30, 40, 41) and the branch defining subtree (30, 40, 41, 50, 51).

Figure 1d presents a more complex scenario where two taxa (*leaf 1* and *leaf 2*, both marked magenta) rearrange independently. In the source tree 1), leaf 1 forms a subtree with leaves 10 and 11, while leaf 2 forms a subtree with leaves 20 and 21. To transform toward the target tree 5), the branches defining the subtrees (1, 10, 11) and (2, 20, 21) are shrunk to zero length and marked with black *x* symbols in tree 2). In tree 3), these zero-length branches are removed, yielding an intermediate strict consensus in which both moving leaves are placed in the same multifurcation outside the pivot edge. The positions of the moving taxa are adjusted to match the target topology while the consensus topology remains unchanged. (tree 3). The new branch defining subtree (1, 20, 21) is expanded in tree 4), and the branch defining subtree (2, 10, 11) is inserted in tree 5). This demonstrates how multitaxon rearrangements are decomposed into sequences of branch collapses and expansions.

For each pair of consecutive trees in the input sequence, Phylo-Movies compares the source tree with the target tree. The shared pivot edge isolates the local conflict between source-unique and target-unique splits, and the affected subtrees are then animated as rooted SPR moves (Bordewich and Semple 2005). Subtrees that remain structurally unchanged between the source and target trees are retained, and only those whose placement differs are animated.

Once the moving subtrees are identified, their relocation toward the target position is animated while the consensus topology remains unchanged. To maintain visual clarity, Phylo-Movies animates these SPR moves sequentially. When a single moving subtree contains multiple internal conflicts, a heuristic decomposes the rearrangement into smaller sequential transitions.

For each rearrangement, the visualization animates the corresponding SPR move by smoothly transitioning from the source tree layout to the target tree layout, producing a sequence of animation frames (30 by default). To minimize extraneous motion, the tool optimizes the leaf ordering of the target tree to match the source layout, ensuring that only subtrees involved in topological changes move. Additional navigation and analysis features are described below.

### Phylo-Movies features and user interface

**Figure 2:**
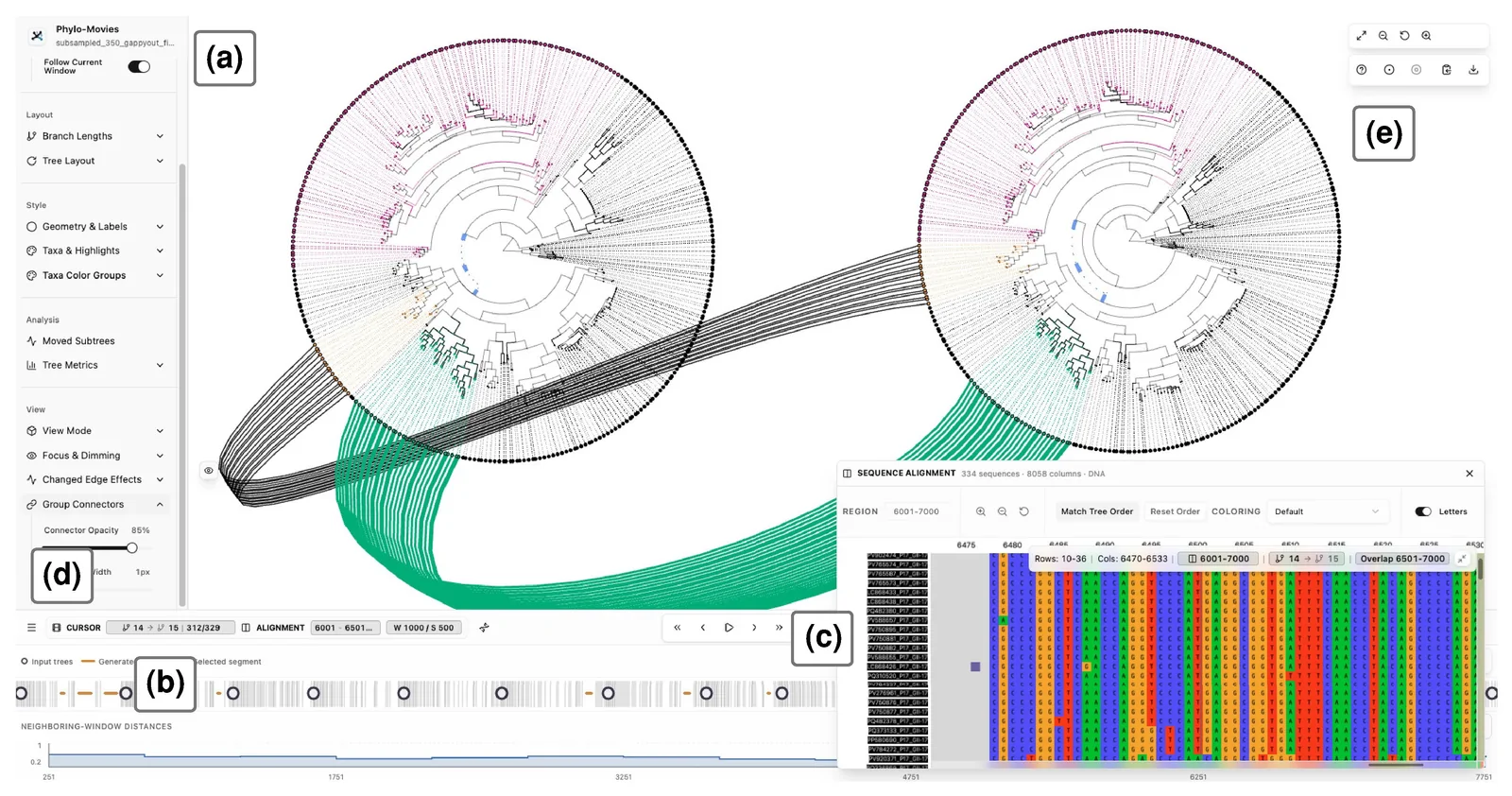
The Phylo-Movies interface for visualizing phylogenetic tree sequences, showing an example analyzing the Norovirus genomes. a) *Tree viewer*: visualizing the phylogeny for the current window/position. At a window position, this is the tree reconstructed for that window; between two window positions, this is the interpolation state between the two adjacent trees. Moving subtrees are linked by the two ribbons between the trees with their pivot edge dash-dotted in blue; the subtree undergoing change in the current step is the upper, more narrow ribbon (here colored black). Leaves can also be colored by user-selected taxon annotation groups, here by polymerase genotype. Phylo-Movies can also show a reference tree, here the target tree, with selected taxa linked by lines. b) *Playback control, progress, and distance panel*: The top row contains control buttons to play, stop, and step through the animation. Below, a progress bar shows the trees of the sequence as circles and the individual transformations as vertical bars; the vertical playhead marks the current position and can be dragged to navigate through the visualization. The distance plot at the bottom helps localize regions of topological change. Users can choose different measures, such as normalized Robinson–Foulds distance, raw weighted RF, or tree-size scale. c) *Alignment viewer*: presents the current region of the underlying alignment, highlighting the active window, with explicit region bounds. When window synchronization is enabled, the alignment position shown will change according to the current tree position. d) *Left control panel*: file/alignment actions and visualization settings (branch-length handling, geometry/layout transforms, taxa coloring, and internal-node/ancestor value display), including the window-synchronization toggle. e) *Top-right canvas controls*: fit, zoom, and reset the tree viewport, and export the current visualization as a still image or recording.

The Phylo-Movies user interface (Fig. 2) consists of several synchronized panels. The *animation and tree viewer panel* (Fig. 2a) displays the transformation between trees, where moving subtrees are highlighted.

The *playback control panel* (Fig. 2b) includes buttons to play, stop, and step through the animation forward and backward. The *timeline chart* (Fig. 2b) tracks the genomic location with a playhead. Each circle represents a tree reconstructed from one sliding window, while each bar between two circles represents the interpolated transition between the two adjacent-window trees. Clicking or dragging the playhead adjusts the current window/position shown in the tree viewer.

The distance plot below the timeline shows a relative RF series (Robinson and Foulds 1979, 1981) between adjacent pairs of input trees. Following the standard split-based definition, each internal branch defines a nontrivial split of the taxa, and RF is the number of splits present in one tree but not the other.

The branch-length-weighted distances are the sum of the absolute differences between the two split lengths (Robinson and Foulds 1979), over all rooted splits with branch lengths that appear in either tree (using 0 for splits that are absent in one of the trees). Users can switch between the relative RF series, the branch-length-weighted RF series, and a tree-size series showing the maximum root-to-tip distance of each input tree.

An *alignment panel* (Fig. 2c) can be chosen to display the genomic alignment synchronized with the current tree, allowing users to associate genome positions with topological changes. The *setup panel* on the left (Fig. 2d) provides configuration options for the visualization and toggles synchronization between the alignment viewer and the displayed tree or interpolation frame. The top-right canvas controls (Fig. 2e) provide viewport actions, such as fitting, zooming, and resetting the tree view, as well as export controls for still images or recordings.

Phylo-Movies also supports side-by-side tree comparison: users can pin any tree from the sequence as a reference (Fig. 2a, right tree), helping to track where subtrees were positioned before and how their placements differ relative to a chosen starting point. Corresponding taxa can be linked between phylogenies to highlight structural differences (as shown in Fig. 2a). Rendering is implemented via WebGL to support interactive tree and alignment display.

The visualization supports several customization options for interpretation. Subtree coloring propagates a leaf color to internal branches only when all descendants share that color, making uniformly colored subtrees visually distinct. Users can load a CSV file with taxon annotations (e.g. genotype, host, or geographic origin) and interactively switch between these grouping schemes. Phylo-Movies also reads and displays support values associated with internal nodes or ancestral branches, like bootstrap support values (Felsenstein 1985), IQ-TREE UFBOOT values (Minh et al. 2013), or SH-aLRT branch-support values (Guindon et al. 2010), such that users can assess whether an animated movement involves well-supported subtrees or mainly low-support, uncertain branches. Node size can be scaled, and highlighted elements—pivot edges, marked subtrees, or structures from previous frames—are rendered with increased radius or thicker outlines so that marked subtrees and active change edges remain distinguishable from baseline coloring.

The tree viewer lets users magnify the tree and move the view across the canvas, and provides controls for font size, branch width, node size, and label spacing. Branch lengths can be displayed proportionally to the input Newick branch lengths, or transformed for readability during animation. By default, branch lengths are square-root transformed and then root-to-tip normalized within each tree, stabilizing the apparent tree size across frames; this display is intended for visual tracking of topology and should not be interpreted as preserving absolute evolutionary scale. Absolute branch-length changes remain available through branch-length-aware distance values.

## Use cases and results

### Recombination-induced topological shifts in norovirus

Recombination drives Norovirus evolution by enabling viruses to acquire novel capsid variants while retaining a functional polymerase, thereby evading host immunity (Bull et al. 2005; Lindesmith et al. 2011; Chhabra et al. 2019). Because the polymerase gene (ORF1) and capsid gene (ORF2) frequently have distinct evolutionary origins, a dual-genotype nomenclature is used; for genogroup II viruses, this is commonly written as GII.*y*[P*x*], where GII.*y* denotes the capsid genotype and P*x* the polymerase genotype (Chhabra et al. 2019). Matching genotypes (e.g. GII.4[P4]) suggest a shared evolutionary history, whereas mismatched combinations (e.g. GII.4[P16]) are consistent with recombinant origins.

To investigate how tree topology shifts across the recombination breakpoint, we used Phylo-Movies to visualize a sliding-window phylogenetic analysis on a global dataset of 334 full-genome Norovirus sequences (8,058-bp alignment) from 32 countries, sampled between 1968 and 2025. Sequences were downloaded from the Nextstrain-curated GenBank dataset (Hadfield et al. 2018), subsampled using Augur (Huddleston et al. 2021), and genotyped using Nextclade (Aksamentov et al. 2021). Using capsid labels from VP1/ORF2 annotations and polymerase labels reduced to their numeric genotype component (P-type), the dataset contains 30 capsid labels and 28 polymerase P-types. Among the 302 sequences with comparable numeric capsid and polymerase assignments, 167 had discordant genotype numbers (55.3%; 50.0% of all 334 sequences).

The sequences were aligned using MAFFT v7.526 (Katoh and Standley 2013) and trimmed using trimAl (Capella-Gutiérrez et al. 2009). We inferred 17 sliding-window trees with the IQ-TREE fast-search option (Minh et al. 2020) under the GTR+G model, using 1,000-bp windows stepped every 500 bp and clipped at the alignment ends. Before Phylo-Movies interpolated the transition between each pair of adjacent-window trees, the resulting tree sequence was midpoint-rooted. Internal branches were annotated using SH-aLRT with 1,000 RELL replicates per window, providing approximate likelihood-ratio support values for the displayed splits (Guindon et al. 2010). We used these SH-aLRT values only to assess support for branch placements; they are not movement counts and do not estimate recombination probabilities. Adjacent-window RF distances were used to locate large topological changes, and Phylo-Movies was used to inspect the corresponding placements and branch-support values (Fig. 2).

At the ORF1/ORF2 junction (approximately at position 5,100 bp), the largest relative RF distance occurred between the windows centered at 5,001 and 5,501 bp (RF=0.565). Branch-length weighted RF distances were also elevated across the following ORF2-side transitions, reaching their maximum between the windows centered at 6,001 and 6,501 bp (weighted RF=21.16). The RF-peak windows had median SH-aLRT branch-support values of 88.0 and 85.8 for the complete split set in each tree, respectively. For the highlighted recombinant groups, ORF1 windows show polymerase-genotype-associated placements, whereas ORF2 windows show capsid-genotype-associated placements.

The most consistent group-level shifts were observed for P16 recombinant lineages. GII.4[P16] recombinants were placed near other P16 sequences in ORF1 but near GII.4 sequences after the transition into ORF2. GII.6[P7] variants (14 sequences), sampled from eight countries across the Americas, Asia, and Africa, were also among the frequent moving-subtree genotype labels, but their P7-associated placements were less clearly resolved than the P16 examples and are therefore treated as exploratory. These patterns illustrate the mosaic origin of recombinant genomes: segments derived from different parental genomic backgrounds form contiguous blocks that associate with distinct taxon sets depending on genomic position. Because the animation decomposes topological transitions into sequential SPR moves, the relocation of these subtrees can be followed step by step. This approach can also be applied to sequences of unknown origin: by including them in the analysis, one can observe which genotype-associated tree regions they join in different genomic regions, helping to assess their polymerase- and capsid-associated placements.

When the data are noisy, numerous small subtree rearrangements may occur and can be tedious to follow individually. The distance plot highlights windows with larger topological changes, while the SH-aLRT branch-support labels help separate well-supported genotype-associated placements from local low-support rearrangements. Across the full tree sequence, 1,616 SPR moves were recorded. Across the 17 input trees, 5,322 internal SH-aLRT branch-support labels were available; the median SH-aLRT branch-support value was 86.0 across all branch-support labels and 91.7 when zero-support labels were excluded, with 3,713 labels (69.8%) at or above 70. When we counted SPR events for moving subtrees that contained at least one sequence with a given genotype label, the genotype labels most often associated with moving subtrees were GII.17[P17], GII.4[P4], GII.4[P31], GII.4[P16], GII.6[P7], and GII.2[P16]. This result indicates that topology changes were not confined to the highlighted P16/P7 recombinants; other genotype labels were also frequently present in moving subtrees, whereas SH-aLRT values describe support for individual branch placements rather than the frequency of these movements.

Phylo-Movies enables direct observation of recombination-associated changes in tree topology: as the sliding window crosses the ORF1/ORF2 junction, polymerase-genotype-associated tree regions dissolve and capsid-genotype-associated tree regions assemble.

### Visual exploration of candidate rogue taxa in bootstrap tree sets

Rogue taxa cannot be reliably placed because conflicting or missing data cause them to shift position frequently across a set of reconstructed trees (Wilkinson 1996). Their presence reduces resolution and support in consensus trees; removing them often yields more informative results. Identifying rogue taxa before final analysis is therefore often important.

When analyzing bootstrap trees, rogue taxa are those that change position across replicates, indicating unstable phylogenetic placement. Phylo-Movies is not intended to replace quantitative rogue-taxon detection methods such as RogueNaRok. Instead, it provides a complementary visual layer for inspecting how candidate rogue taxa move across bootstrap replicates: whether they jump diffusely across the tree or repeatedly alternate between a small number of competing placements. To demonstrate this, we used two MSAs from the RogueNaRok dataset (Aberer and Stamatakis 2013a, 2013b): dataset 24 (24 taxa, 14,190 sites), corresponding to the palaeognath mitochondrial alignment analyzed by Phillips et al. (2010), in which *Ostrich* has been identified as a rogue taxon due to unstable placement across bootstrap replicates; and dataset 125 (125 taxa, 29,149 sites), whose taxa are anonymized (Seq1, Seq2, Seq3, etc.) and were contributed for benchmarking without disclosure of species identities, which we analyzed to evaluate performance on a dataset larger than dataset 24.

To order the resulting trees in a reproducible trajectory that facilitates visual tracking of positional changes, we sorted replicates by their similarity in state counts to the original (nonresampled) alignment. For each replicate *b*, we computed the state-count vector $c_{b}=(n_{A},n_{C},n_{G},n_{T},n_{\mathrm{ambiguous}/\mathrm{gap}})$ and measured its deviation from the original alignment using the Euclidean distance:


(1) $$d_{b}=\sqrt{\sum_{i\in \{A,C,G,T,\mathrm{ambiguous}/\mathrm{gap}\}}(c_{b,i}-c_{\mathrm{original},i}{)}^{2}}$$

where $c_{b,i}$ and $c_{\mathrm{original},i}$ denote the counts of state *i* in bootstrap replicate *b* and in the original MSA, respectively. Replicates were sorted by increasing $d_{b}$. Bootstrap replicates have no intrinsic biological order; any linear arrangement imposes a trajectory for visualization. This does not alter the inferred trees themselves but determines which pairs of trees are compared consecutively for the animation.

Because this order is based on the nucleotide composition of the replicate alignments rather than tree-to-tree distances, adjacent trees are not necessarily close in topology or branch length. As an order-sensitivity check, we also arranged the same 200 trees by a rooted nearest-neighbor path optimized using the 2-opt algorithm (Croes 1958), improving the weighted RF distance (Robinson and Foulds 1979) between adjacent inferred trees while breaking ties using relative RF distance and composition order. The branch-length-weighted RF distance was used first, relative RF distance second, and the original composition-ranked order only as a final tie-breaker.

Bootstrap replicate alignments were generated with RAxML raxmlHPC -f j (Stamatakis 2014), and the maximum-likelihood tree for each replicate alignment was inferred using IQ-TREE 3 (Wong et al. 2026) with default tree-search procedure and SH-aLRT branch support (1,000 RELL replicates). For the Phylo-Movies examples discussed here; however, the displayed internal branch labels were split-frequency support values calculated across the same 200 inferred bootstrap-replicate trees, ordered by ascending composition distance. These split-frequency labels report support for individual splits; the SPR movement counts below are computed separately from the midpoint-rooted ordered tree sequences. Before interpolation, each 200-tree sequence was midpoint-rooted, and for each ordered tree sequence we counted how often taxa or subtrees were moved during the SPR decomposition. Thus, branch labels report split support, whereas these movement counts report how often taxa or subtrees changed placement across the ordered tree sequence.

We asked whether taxa identified as rogue by RogueNaRok also appeared among subtrees that were moved repeatedly when Phylo-Movies animated this trajectory of bootstrap trees (Aberer and Stamatakis 2013b). RogueNaRok identifies rogue taxa by optimizing the relative bipartition information criterion (RBIC), which measures the overall support across a bootstrap tree set relative to the maximum achievable support. In contrast, Phylo-Movies does not compute a support-based criterion; instead, it decomposes differences between trees into sequential SPR moves and highlights the subtrees that change position across replicates. Counting how often taxa or subtrees are moved during the SPR decomposition therefore provides an exploratory view of placement uncertainty: it shows which taxa or subtrees repeatedly participate in SPR moves and where those moves occur in the ordered tree sequence, while support-based filtering and rogue-taxon scoring remain tasks for dedicated quantitative methods.

In the 24-taxon dataset, based on 200 bootstrap trees, RogueNaRok reported an increase in RBIC from 0.828810 to 0.853333 upon removal of *Ostrich*. Correspondingly, the singleton moving subtree containing *Ostrich* was the subtree moved most often during the SPR decomposition, ranking 1st out of 27 repeatedly moved subtrees (79 SPR moves; 19.60% of 403 SPR moves; above 96.3% of moving subtrees by number of SPR moves). The complete tables are available in our GitHub repository and can also be accessed directly through the example datasets provided within Phylo-Movies.

In the 125-taxon dataset, also comprising 200 bootstrap replicates, RogueNaRok reported an increase in RBIC from 0.958484 to 0.958549 upon removal of *Seq112*. When counting how often taxa or subtrees were moving during the SPR decomposition, the singleton moving subtree containing *Seq112* ranked 2nd out of 44 repeatedly moved subtrees by SPR-move count, tied with two other subtrees (91 SPR moves each, i.e. 8.47% of 1,074 SPR moves; above 90.9% of moving subtrees by number of SPR moves).

The weighted-RF sensitivity analysis showed that the composition-ranked trajectory differs from a tree-distance path: the two orders shared only one of 199 adjacent pairs in dataset 24 and two of 199 in dataset 125. In dataset 24, total adjacent weighted RF fell from 107.62 to 56.68 and the SPR decomposition from 403 to 166 moves; *Ostrich* remained the most frequent singleton mover (38 moves). In dataset 125, total adjacent weighted RF fell from 180.87 to 153.52 and the SPR decomposition from 1,074 to 661 moves; *Seq112* ranked first (70 moves). Thus, exact SPR counts and ranks depend on the imposed order; however, the RogueNaRok-identified candidates remained prominent under the independent tree-distance-based ordering.

Together, these results show that Phylo-Movies provides an interactive visual approach for exploring order-dependent SPR-move patterns of candidate rogue taxa. RogueNaRok and Phylo-Movies measure different properties: RogueNaRok optimizes a support-based RBIC criterion, whereas Phylo-Movies reports repeated topology-changing movements across an ordered tree sequence.

## Discussion

We have shown two use cases where visualizing tree sequences reveals evolutionary patterns that would be difficult to obtain by manual inspection. We illustrated this approach by inspecting a recombination breakpoint that underlies the differences observed between Norovirus genotypes and by visualizing placement changes of candidate rogue taxa associated with placement instability.

Beyond these two use cases, Phylo-Movies can be applied to other empirical or simulated tree sequences where users need to inspect which taxa or subtrees change placement. For example, in population simulations with recombination using tools such as the tskit framework (Kelleher et al. 2016), Phylo-Movies can visualize the resulting tree sequence and show how recombination events alter genealogical structure along the genome. Similarly, if an analysis indicates topology changes or candidate recombination breakpoints in a genomic region, Phylo-Movies can be used to visualize which subtrees or taxa change position in the corresponding tree sequence. There are no restrictions on the origin of the underlying MSA from which the trees were inferred (e.g. bacterial or eukaryotic taxa), nor on the process that generated the resulting tree sequence.

Phylo-Movies can also be used to investigate additional aspects of tree variation along the underlying genomic sequence, including changes in phylogenetic resolution, instability of root or outgroup placement, branch-length variation, and other topology shifts not necessarily caused by recombination. Because Phylo-Movies accepts multifurcating trees, users can observe how phylogenetic resolution varies across genomic regions—for instance, identifying windows where certain subtrees collapse into multifurcations due to insufficient informative sites, which may guide the choice of window size or indicate regions of genuine evolutionary uncertainty.

Similarly, one can check whether an outgroup used for rooting the trees remains stable, whether a multifurcation occurs near the root (indicating loss of phylogenetic signal), or whether the outgroup changes position across genomic regions (showing positional instability). Both outcomes—loss of resolution and positional instability—show that the outgroup does not consistently root the tree across all genomic regions. Finally, Phylo-Movies is also able to visualize the changes in branch lengths along the genome, even when tree topology remains unchanged across the analyzed region. Such changes may be caused by selection effects, which are sometimes misinterpreted as recombination events in purely distance-based analyses (e.g. Worobey et al. 2002).

For methodological diagnostics, users can compare trees inferred from the same data under different analysis settings, such as alternative substitution models or inference programs, and inspect which subtrees are rearranged. When developing or analyzing tree search strategies, Phylo-Movies can be used to study the search trajectory through tree space, identifying which subtrees remain stable early versus those that continue rearranging until the final iterations. Because the animation exposes otherwise abstract tree rearrangements as visible SPR moves, Phylo-Movies can also be used as an educational tool in teaching settings and hands-on workshops.

When analyzing tree sequences with many taxa, numerous trees, and frequent rearrangements, the distance plot helps locate regions of topological change so that users need not watch the entire animation to find them. The practical limit is both computational and perceptual. WebGL rendering supports interactive exploration of hundreds of taxa on typical laptops, as illustrated by the 334-taxon Norovirus dataset and the 125-taxon RogueNaRok example, but label readability, screen or projector resolution, and the number of intermediate transitions become limiting factors as datasets grow.

For particularly large or complex datasets, users can select a contiguous range of trees, thin the tree sequence to include only every *k*th tree, hide labels, or focus on subtrees that are moved repeatedly during the SPR decomposition. Although only 17 trees were inferred in the Norovirus analysis, the numerous and complex rearrangements between consecutive windows required 6,391 interpolated intermediate trees for visualization. Larger tree sequences require more intermediate states because each rearrangement must be decomposed into sequential SPR moves, which may reduce responsiveness and make individual SPR moves harder to track in highly complex analyses.

Like FigTree (Rambaut 2006) and DensiTree (Bouckaert 2010), Phylo-Movies reads trees in standard Newick format. However, whereas FigTree displays static images of individual trees and DensiTree summarizes a set of trees by overlaying them, Phylo-Movies animates the topological changes between consecutive trees. In addition, Phylo-Movies can display two trees side by side, linking corresponding taxa with connecting lines, similar to a tanglegram (Page 2002; Scornavacca et al. 2011), so that users can easily track the source and target placements of moving subtrees during the animation. Phylo-Movies also provides functionality for alignment inspection, sliding-window tree inference, taxon-annotation-based coloring, and video export, supporting the interpretation of topological changes within a tree sequence.

## Data Availability

All sequence alignments, phylogenetic trees, taxon annotations, result tables, and code used in this study are available in the Phylo-Movies repository at https://github.com/enesBerkSakalli/phylo-movies. The public software landing page and interactive online demos are available at https://enesberksakalli.github.io/phylo-movies/. Phylo-Movies is released under the MIT License and includes a standalone desktop application. For the Norovirus example, corresponding GenBank accession numbers are provided in the repository accession table; accession information for the other examples is provided there as well. The underlying transformation engine is implemented in the BranchArchitect framework, which computes and decomposes topological differences between trees into sequences of rooted subtree prune-and-regraft (SPR) moves. The BranchArchitect source code is available at https://github.com/EnesSakalliUniWien/BranchArchitect. Demonstration videos are available as: *Installation and small example* (https://vimeo.com/1199476378); *Synced MSA Example* (https://vimeo.com/1199476382); *Norovirus Example* (https://vimeo.com/1199476534); *Phylo-Movies 125 Seq Example* (https://vimeo.com/1199487394); and *24 Taxa Rogue Taxon Example* (https://vimeo.com/1199495473).
